# Female couples undergoing assisted reproduction - choices and the importance of pregnancy and genetics

**DOI:** 10.5935/1518-0557.20230007

**Published:** 2023

**Authors:** Pedro Brandão, Nathan Ceschin, Bodil Sandvik, Stefania Paolelli, Jakob Doblinger, Sérgio Reis-Soares, Ricardo Sousa-Santos, José Bellver

**Affiliations:** 1 Department of Reproductive Medicine, IVIRMA Valencia Plaza de la Policia Local 3, 46015, Valencia, Spain; 2 Faculty of Medicine, University of Porto Alameda Prof. Hernâni Monteiro, 4200-319 Porto, Portugal; 3 Department of Reproductive Medicine, Ginemed Porto, Avenida da Boavista 1243, 4100-130 Porto, Portugal; 4 Department of Reproductive Medicine, Feliccità Fertility Institute R. Conselheiro Dantas, 1154 - Prado Velho, Curitiba - Paraná, 80220-191, Brazil; 5 Department of Reproductive Medicine, Feliccità Fertility Institute R. Conselheiro Dantas, 1154 - Prado Velho, Curitiba - Paraná, 80220-191, Brazil; 6 Medicaly Assisted Reproduction Centre, Hospital da Senhora da Oliveira, R. dos Cutileiros 114, 4835-044 Guimarães, Portugal; 7 IVI Foundation, Instituto de Investigación Sanitaria La Fe, Valencia, Spain Avenida Fernando Abril Martorell, 106 - Biopolo, Torre A, Planta 1ª 46026 Valencia, Spain; 8 Department of Pediatrics, Obstetrics and Gynaecology, University of Valencia. Av. Blasco Ibáñez, 15. 46010 Valencia, Spain

**Keywords:** assisted reproductive technology, female couple, genetics, pregnancy, ROPA

## Abstract

**Objective:**

To evaluate female couples’ reproductive choices, the importance given to genetics and pregnancy and their expectations regarding mother-child relationship.

**Methods:**

Observational study based on an anonymous survey applied to 217 patients during 2021. The survey was given to female couples under reproductive treatment in a private fertility clinic. The outcomes were divided into 3 main groups: the choice of their reproductive treatment, motherhood and biological links, and their plans for future reproductive treatments.

**Results:**

Most patients found it easy to choose their treatments and roles. The choice was mainly driven by success rates, costs, and simplicity, except for ROPA for which sharing biological motherhood was the main reason. Most couples consider genetics and pregnancy important but, in the end, they believe they will have a similar connection to their child, regardless of the role played. In the future, some couples consider doing the same treatment while others consider inverting roles.

**Conclusions:**

Most female couples have no difficulty when it comes to choosing a reproductive treatment or role to play, mainly based on costs, success rates and the possibility of sharing biological motherhood with the ROPA method. These patients give great importance to genetics and pregnancy, but they expect a similar connection to their child regardless of the type of treatment and the roles played.

## INTRODUCTION

Worldwide, the access of same-sex couples to medically assisted reproduction has been gradually expanding (Ethics Committee of American Society for Reproductive Medicine, 2013). With respect to female couples’ reproduction, both intrauterine insemination (IUI) and *in vitro* fertilization (IVF) with donated sperm are viable options. The former is simpler, cheaper, requires less medication and is associated with fewer risks. On the other hand, IVF has higher rates of success and enables the creation of surplus embryos, which can be used in subsequent treatments in cases of failure or desire of additional children from the same donor (Brandão *et al*., 2022a). During the last decade, a new method has been used to enable both members of female couples to be biological mothers of the same child – the ROPA method (in Spanish: *Recepción de ovocitos de Pareja*; in English: Reception of oocytes from partner) also known as lesbian shared IVF or co-IVF (Yeshua *et al*., 2015; Núñez *et al*., 2021). With this method, the oocytes of one of the patients (“donor” or “genetic mother”) are fertilized with donated sperm and the embryo is transferred to the other member of the couple’s uterus (“recipient” or “gestational mother”). This method enables both members of female couples to have an active role in the generation of a child (Marina *et al*., 2010; Bodri *et al*., 2018).

Assisted reproductive techniques are not risk-free and some concerns have been raised regarding if it would be ethical to submit a biologically fertile woman to these treatments. However, it is generally accepted that, whenever it corresponds to the patient’s desire, the importance of these treatments to the individual’s wellbeing overwhelms potential damages (Fiske & Weston, 2014; Brandão *et al*., 2022b).

As a consequence of progressive changes in national legislations worldwide, an increase in the demand for reproductive treatments by female couples has been observed (Fiske & Weston, 2014). Apart from the need of donated sperm, up to 40% of them may have a fertility disorder (Power *et al*., 2020; Brandão *et al*., 2022a). Some studies report that the medical and surgical background of lesbian patients who search for assisted reproduction is similar to those of the general population, concerning medical and surgical history (Kim *et al*., 2020). In addition, the outcomes of treatments do not appear to be influenced by sexual orientation (Nordqvist *et al*., 2014; Soares *et al*., 2019). Although almost all of the female couples do not have history of infertility, more than half of the patients end up undergoing IVF instead of intrauterine insemination, due to fertility disorders discovered during medical workup (Power *et al*., 2020). Interestingly, despite the availability of ROPA, previous studies showed that more than 75% of female couples choose a “one parent” (or “single-way”) reproductive treatment (Brandão *et al*., 2022a).

Little has been published on what motivates female couples in their choice of treatment and roles to play, as well as the importance of a biological connection to their offspring. Concerning the ROPA method, even though the first study about it has been published more than 10 years ago, few studies have been developed since then (Marina *et al*., 2010). Despite the paucity of information, the outcomes of this technique are reassuring (Brandão *et al.,* 2022c).

This study aims to clarify the reason leading female couples’ choices on their reproductive pathway, the importance given to genetics and pregnancy as a biological connection to a child, and their expectations regarding subsequent mother-child connection.

## MATERIALS AND METHODS

### Study design

This is an observational study based on an anonymous survey applied to female couples under reproductive treatments. The study was approved by the local Institutional Review Board, under the code 2012-VLC-104-PB.

### Study sample

Inclusion criteria were female couples (biological gender) under any fertility treatment at IVI Valencia during 2021, including IUI, IVF (with own or donated oocytes/embryos) and the ROPA method. Exclusion criteria were patients undergoing treatments as single, transgender patients, patients who could not read any of the languages in which the survey was available, patients who had already participated or who refused to answer the survey.

### Survey

The survey was originally written in English (supplement. 1). It was a paper-based questionnaire with 2 pages, divided into 5 sections: personal data, previous motherhood, current treatment, importance of pregnancy and genetics and future reproductive treatments. Afterwards, the survey was translated to French, Italian, Portuguese, and Spanish by native or proficient speakers. The final version of each language was rechecked by a second proficient speaker to assure its exact translation.

Patients who had already started a fertility treatment were invited to participate in the study, after explanation and delivery of an information sheet.

Each patient was given a copy of the questionnaire in her language of choice. At the beginning of the questionnaire the patients were asked to give their informed consent to participate.

Completed questionnaires were anonymously deposited in a locked box. When both members of the couple were present, the questionnaires were stapled together.

### Data analysis

The answers were compiled in an SPSS® database for statistical analysis. Means and proportions were calculated for continuous and categorical variables, respectively. Depending on the variable in question, the following comparisons were made: patients on single-way versus ROPA treatments, patients with an active role (“active patients”, which means patients being gestational or genetic mothers-to-be or both) versus patients with no active role (“passive patients” – the partners of the patients being treated), genetic versus non-genetic mothers-to-be and gestational vs non-gestational mothers-to-be. After visually assessing normality of the continuous variables, parametric (T-test) and non-parametric (Mann-Whitney) tests were used to compare normally and non-normally distributed variables, respectively. The Chi-square test was used to compare categorical variables. A significance level of 0.05 was used. Missing data were excluded.

The outcomes were divided into 3 main groups: those regarding reproductive treatments (difficulty and reasons for choosing treatment and roles), those regarding motherhood and biological links (the desire of being mother, the importance of pregnancy and genetics and the expected mother-child connection) and those concerning future reproductive treatments (the desire to have more children and which reproductive treatment to choose in the future). The outcomes concerning reproductive treatments were compared between patients undergoing single-way treatments and ROPA, while the other variables were compared considering all groups.

## RESULTS

A total of 217 surveys were obtained. In 17 of the 117 participating couples, only one member of the couple completed the questionnaire.

No differences were found between groups concerning age, years of relationship, level of education and previous motherhood (Table 1).

**Table 1:** Baseline characteristics of the sample, compared between single-way and ROPA patients, patients with and active *vs.* passive role, genetic *vs.* non-genetic mothers, and gestational *vs.* non-gestational mothers.

		All	Single-way vs. ROPA			Active vs. passive role			Genetic vs. non-genetic mother			Gestational vs. non-gestational mother		
			Single-way	ROPA	P	Active	Passive	P	Genetic	Non-genetic	P	Gestational	Non-gestational	P
Age (mean)		35.8	34.8	36.2	0.08	35.4	36.6	0.15	35.2	36.4	0.11	35.5	36.1	0.41
Years of relationship (mean)		7.8	7.3	8.0	0.31	7.7	8.0	0.64	7.8	7.7	0.94	7.8	7.8	0.98
Level of education (%)	Basic	2.3	1.6	2.6	0.48	1.4	4.0	0.35	0.9	3.7	0.71	1.7	3.0	0.60
	Highschool	23.7	23.4	23.8		23.6	23.9		22.6	24.8		24.3	23.0	
	Graduation	40.0	39.1	40.4		41.7	36.6		41.5	38.5		43.5	36	
	Master	25.8	34.4	26.5		29.9	26.8		30.2	27.5		27.0	31.0	
	PhD	5.1	1.6	6.6		3.5	8.5		4.7	5.5		3.5	7	
Previous children (yes - %)		39.6	39.2	40.6	0.88	40.4	38.0	0.77	38.0	41.3	0.68	41.0	38.0	0.68

**Figure 1:**
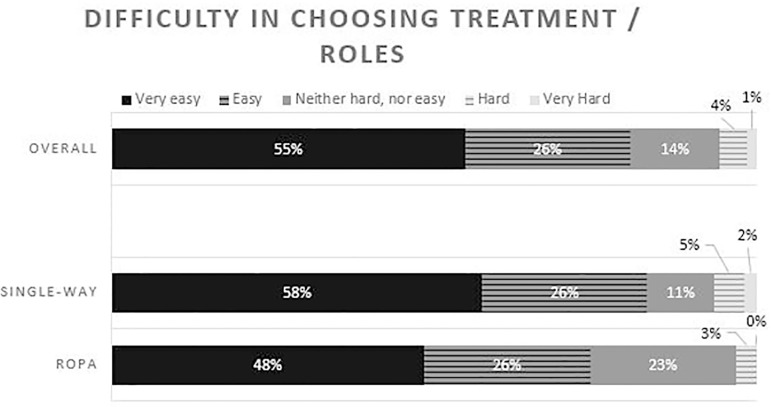
Answers to the question - “How difficult was it to decide who is going to get the treatment or what roles you are you going to play? - The overall results and the comparison between single-way and ROPA patients (*P*=0.151).

More than three quarters of the inquired patients found it easy or very easy to choose treatment and roles to play, and no differences were found between single-way and ROPA patients (Figure 1).

**Figure 2:**
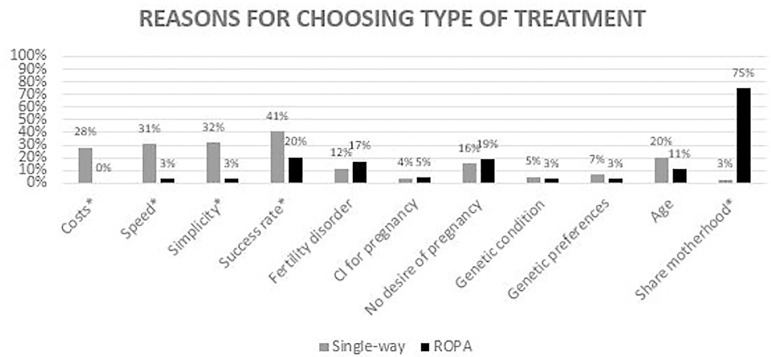
Reasons reported for choosing a single-way or a ROPA treatment. CI: contraindication, *: *P*<0.05.

The most reported reasons for choosing single-way treatments were the success rates, simplicity, speed, and costs. As for ROPA, the vast majority (75%) of the patients reported the possibility of sharing biological motherhood as a reason for undergoing this treatment (Figure 2).

**Figure 3:**
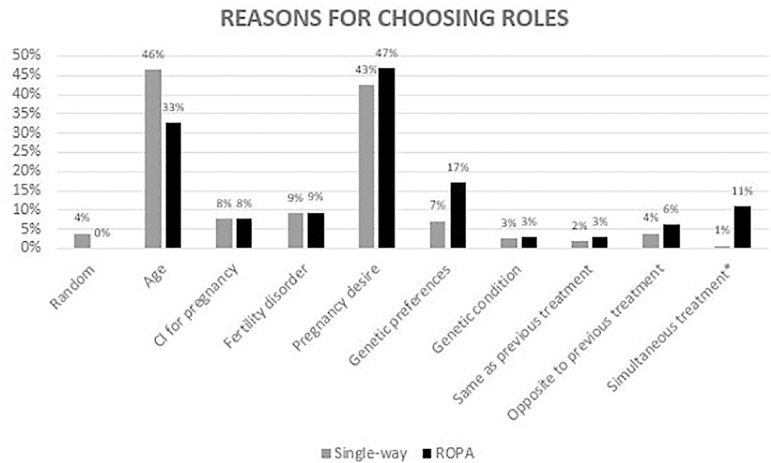
Reasons for choosing roles – active/passive role for single-way treatments, gestational/genetic mother for ROPA treatment. CI: contraindication; *: *P*<0.05.

Regarding the reasons for choosing roles (active/passive if single-way, pregnant/genetic/both/none if ROPA), the main reasons pointed out were the preference of one of the patients for being pregnant (43.8%) and age (42.4%). In the specific case of ROPA, the preference for one’s genetics (17.2%) and the possibility of simultaneously perform both roles (10.9%) were also relevant (Figure 3).

**Figure 4:**
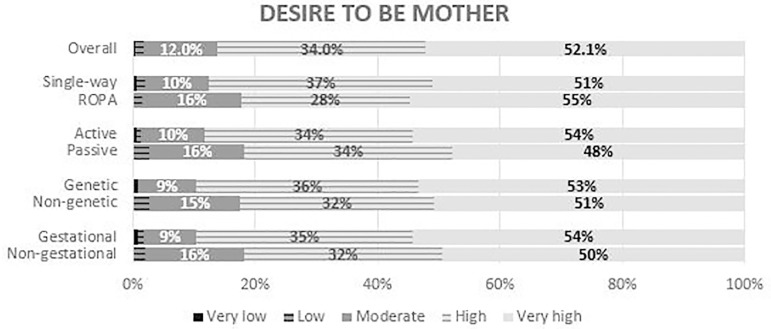
Answers to the question - “How would you rate your wish to be a mother?” – the overall results and the comparison between single-way and ROPA patients (*P*=0.59), active and passive patients (*P*=0.49), genetic and non-genetic mothers (*P*=0.22), and gestational and non-gestational mothers (*P*=0.37).

A large majority of the patients responded that they had a strong or very strong desire to become a mother, regardless of the group (Figure 4).

**Figure 5:**
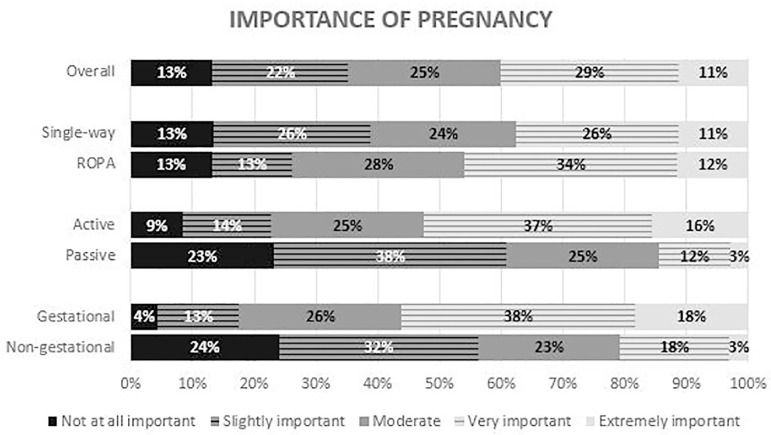
Answers to the question - “How important is it to you to be pregnant of your children?” – the overall results and the comparison between single-way and ROPA patients (*P*=0.35), active and passive patients (*P*<0.01) and gestational and non-gestational mothers (*P*<0.01).

**Figure 6:**
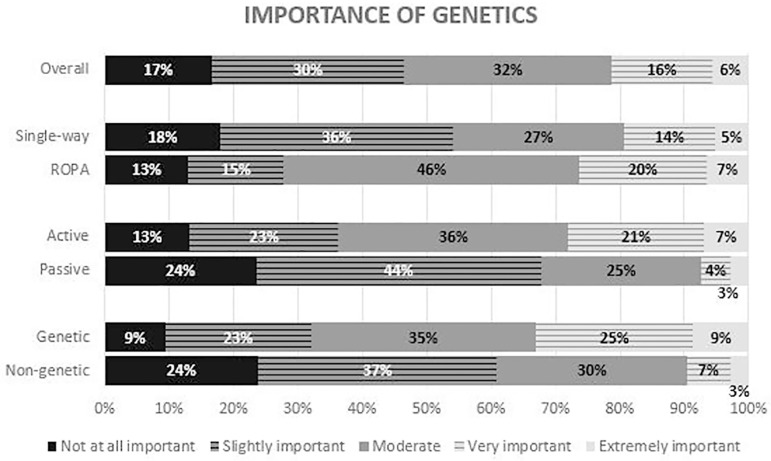
Answers to the question - “How important is it to you that your children have your genes?” – the overall results and the comparison between single-way and ROPA patients (*P*=0.1), active and passive patients (*P*<0.01), and genetic and non-genetic mothers (*P*<0.01).

Forty percent of the patients said pregnancy was very or extremely important; while 26.2% said it was only slightly important or not important at all. Mothers with an active role, including gestational mothers, tended to ascribe more importance to pregnancy. However, no differences were observed between ROPA and non-ROPA patients (Figure 5). In contrast, fewer women attributed much or extreme importance to genetics, and more reported little or no importance, compared to pregnancy. Again, women with an active role, in particular the genetic mothers, tended to attribute more importance to genetic links (Figure 6).

**Figure 7:**
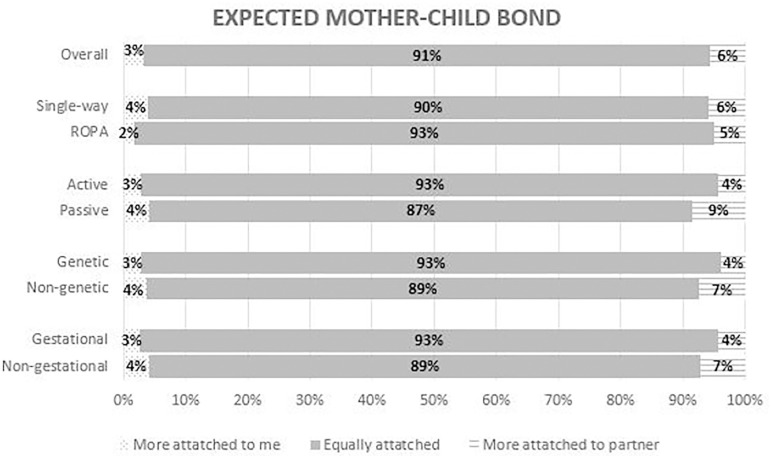
Answers to the question - “How attached do you think your child will likely be to you and your partner?” – the overall results and the comparison between single-way and ROPA patients (*P*=0.67), active and passive patients (*P*=0.37), genetic and non-genetic mothers (*P*=0.47), and gestational and non-gestational mothers (*P*=0.54).

Most patients (91.1%) are confident that their offspring will have a similar bond to themselves and their partner. No differences were observed at this level between ROPA and non-ROPA patients, as well as patients with or without any kind of active role (Figure 7).

**Figure 8:**
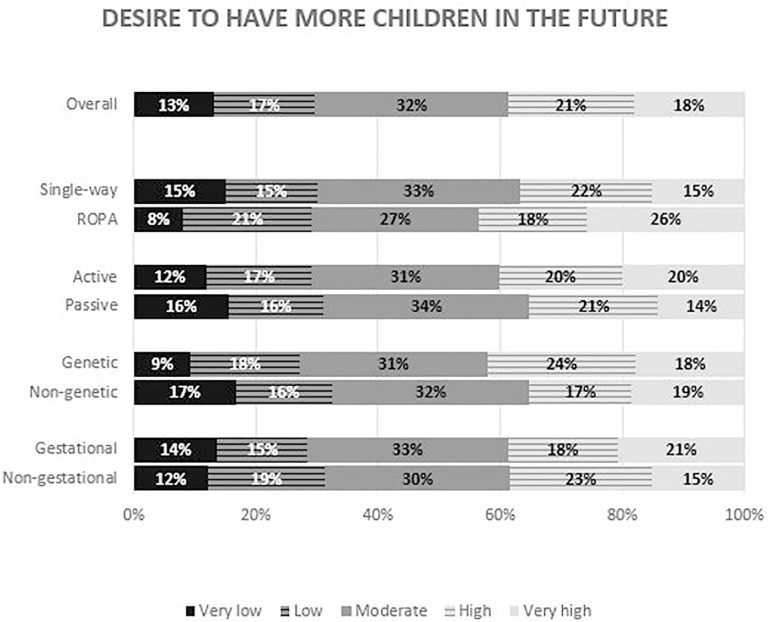
Answers to the question - “How would you rate your wish to have more children after this treatment?” – the overall results and the comparison between single-way and ROPA patients (*P*=0.19), active and passive patients (*P*=0.78), genetic and non-genetic mothers (*P*=0.42), and gestational and non-gestational mothers (*P*=0.66).

Regarding the desire to have more children in the future, opinions diverge in a similar pattern among groups (Figure 8). Almost half of the patients said they would undergo another treatment playing the same roles; while 37.7% said they would invert roles. No differences were found between groups. Interestingly, 27.1% of the women undergoing a ROPA treatment said they would opt for a single-way treatment in the future; but only 10.1% of women doing a single-way treatment said they would switch to ROPA (Table 2).

**Table 2:** Proportion of patients according to the treatment they would choose in the future – overall results and the comparison between current single-way and ROPA patients.

	Overall	Single-way vs. ROPA		
		Single-way	ROPA	p
Same direction (%)	47.3	53.4	32.2	0.11
Opposite direction (%)	37.7	36.5	40.7	
Change single-way to ROPA or vice-versa (%)	15.0	10.1	27.1	<0.01

## DISCUSSION

According to previous literature, around 25% of the female couples opt for a single-way treatment (Brandão *et al*., 2022a). In our study, most patients reported no difficulties in choosing the type of treatment and roles. It seems clear that choices regarding reproductive treatment were not a problem for most couples.

In our sample, most patients chose treatments for their success rates, costs, and simplicity, except for ROPA for which sharing biological motherhood was the main reason. These findings are in line with published literature (Carpinello *et al*., 2016; Brandão *et al*., 2022a). The same applies to the choice of roles to be played. The desire to become pregnant, age and genetic preferences were pointed by the participants as the main factors. However, one should keep in mind that these questions were retrospective, since participants were already under treatment at the time of the study and main decisions were already made.

As expected, most respondents showed a high or very high desire to be a mother (Bos, 2003). The answers to this question were quite similar between ROPA and single-way patients, patients with and without an active role, genetic and gestational mothers. Hence, we infer that the desire to be a mother seems to be unrelated to the patients’ active involvement in her own treatment.

Biological motherhood may be defined by gestation or genetics, and the importance given to each factor varies considerably (Di Nucci, 2016; Thornton & Resetkova, 2021). In our study, most patients say that pregnancy is very or extremely important. Based on our data, gestational mothers seem to give more importance to pregnancy. As mentioned previously, the desire for pregnancy seems to be an important factor for a woman to choose to take on this role. A slightly smaller percentage of patients ascribe a great or extreme importance to genetics. Likewise, genetic mothers tend to attribute more importance to this factor. These results suggest that both pregnancy and genetics seem to have a preponderance in the choice of reproductive treatment, but female couples tend to give more importance to the former.

Based on heteronormative families, children create bonds with various caregivers, but the type of connection they form with their mother, father or others varies considerably (Pelka, 2009). Some previous literature reports feelings of jealously between female couples when they become mothers, based on the fear of being neglected by a non-biological child (Pelka, 2009). In addition, some authors argue that children tend to have a main attachment figure (Ciano-Boyce & Shelley-Sireci, 2002). Nevertheless, other studies show that female couples are able to reconcile the various parenting roles and achieve a balance in their relationship with their children (Bos *et al*., 2004; 2007; Dahl & Malterud, 2015). Based on our results, despite the different positions regarding the importance of genetics and pregnancy, most patients believe they will have a similar bond to their children, regardless of the type of treatment or role.

Concerning future reproductive plans, answers were quite diverse between participants. In addition, a much higher percentage of patients in the ROPA group said they would switch to a single-way treatment than the opposite. This unexpected finding could be interpreted as dissatisfaction with the ROPA method, its costs and complexity, or simply as evidence that women who already biologically contributed to motherhood are more open to simpler treatments in the future. In addition, clinical criteria, such as age or fertility issues may also justify the change. These results open new doors for future research regarding patients’ post treatment satisfaction with the ROPA method.

The main limitation of this study is the somewhat limited sample size. It is also important to bear in mind that our study is limited to a private setting. In fact, this is practically unavoidable since virtually all ROPA treatments are carried out in a private setting.

## CONCLUSION

According to our study, female couples consider they had no difficulty in choosing their reproductive treatment or roles to play. The main motivations for these choices were speed, success rate, simplicity, and cost. Most couples opting for ROPA do so to share biological motherhood.

The importance given to pregnancy and genetics varies considerably between patients, with gestation seeming to be of slightly greater importance. The importance attributed to pregnancy and genetics is higher in patients with an active role, particularly for gestational and genetic mothers, respectively. Nevertheless, most mothers believe that the bond with their children will be similar for both.

Future reproductive plans may very among female couples under treatment, including redoing treatments with the same or opposite roles. An important proportion of ROPA patients would change to single-way treatment in the future.

## MADRES MÍAS PROJECT CHOICES OF FEMALE HOMOSEXUAL COUPLES UNDERGOING ASSISTED REPRODUCTION

### INTRODUCTIÓN

The objective of this questionnaire is to evaluate the reproductive choices of female couples undergoing assisted reproduction. The questionnaire is 2 pages long and is expected to take about 2 minutes to complete.

It is completely anonymous. It has no identification or code and will be deposited randomly along with the other answered questionnaires. Your participation is voluntary. Data will be stored, analyzed and the results of the study may be presented at scientific meetings and / or published in scientific journals, scientific or social communication media.

By completing this survey, you are accepting that your answers will be processed according to the above.

### CONSENT

Do you accept the collection, storage and processing of your answers, as well as the presentation and publication of the results of the study, according to what was previously stated in the introduction?

**Yes** □

**No** □

### CONCEPTS

- **Intrauterine insemination (IUI):** Insemination of sperm into the uterus.
- ***in vitro* Fertilization (IVF):** oocyte (eggs) pick-up, fertilization sperm in the laboratory and transfer of an embryo to the uterus
- **ROPA (also known as shared IVF):** IVF method for female couples in which one of the women gives the oocyte (genetic/donor mother) and the other receives the embryo and becomes pregnant (gestational/recipient mother).
- **Gestational / recipient mother:** woman who will receive the embryo and become pregnant.
- **Genetic / donor mother:** woman who will provide the oocytes (eggs) so that her partner becomes pregnant with them.
- **Reciprocal ROPA:** both play both roles at the same time (donating oocytes to each other reciprocally and simultaneously)
- **Reverse ROPA:** opposite roles compared to a previous ROPA treatment (the previous gestational mother will now be the genetic mother and vice versa)

### 1 – PERSONAL DATA

**1A - Age:**__________

**1B - Level of education:**

□ No studies

□ Graduation (university)

□ Elementary school

□ Master

□ High school

□ PhD

**1C – How long have you been together** (years as a couple – sum of years as girlfriends and spouses)?:

__________________________________________________________________________________________________

### 2 - CHILDREN

**How many children did you have with each of the following methods?** (Indicate the number, e.g.: 0, 1, 2,….)

2A – Spontaneous pregnancy (with a previous or current male partner, with a friend or acquaintance) ______________

2B - Adoption ___________

2C – Step adoption (adoption of partner’s child) ___________

Intrauterine insemination (IUI) / in vitro fertilization (IVF):

2D –You have undergone insemination or IVF with your own eggs __________

2E - Your partner has undergone insemination or IVF with her own eggs __________

2F - You have undergone IVF with eggs from a donor __________

2G - Your partner has undergone IVF with eggs from a donor __________

ROPA (shared IVF):

2H - Genetic mother (you donated the eggs; your partner became pregnant with your eggs)

2I - Gestational mother (you became pregnant with your partner’s eggs) _____________

### 3 - CURRENT TREATMENT

**3A - Which of the following best describes your CURRENT TREATMENT?** (Please select **ONE** correct answer from “a” to “i”) Intrauterine insemination / in vitro fertilization (IVF)

a. □ You are undergoing insemination or IVF with your own eggs
b. □ Your partner is undergoing insemination or IVF with her own eggs
c. □ Both are undergoing insemination or IVF each with your own eggs
d. □ You are undergoing IVF with eggs from a donor
e. □ Your partner is undergoing IVF with eggs from a donor
f. □ Both are undergoing IVF with eggs from a donor ROPA (shared IVF)
g. □ Genetic mother (you are providing the eggs; your partner is going to get pregnant with your eggs)
h. □ Gestational mother (you are going to get pregnant with your partner’s eggs)
i. □ Reciprocal ROPA: exchange of eggs and both are supposed to get pregnant with the same treatment

**3B - What were the main reasons for choosing this type of treatment (choosing between IUI, IVF and ROPA)?** (choose **ALL** the correct answer (s))

a. □ Costs
b. □ Time (speed of the process)
c. □ Simplicity / complexity of the process
d. □ Probability of success
e. □ Medical issues involving fertility (yours or your partner’s)
f. □ Medical issues partially or absolutely contraindicating pregnancy (yours or your partner’s)
g. □ Genetic disease (yours or your partner’s)
h. □ Genetic preferences (preference for your or your partner’s genetics)
i. □ Age - your age
j. □ Age - your partner’s
k. □ Biologically sharing motherhood
l. □ One of you does not want to get pregnant
m. □ Other:_________________________________________

**3C - How difficult was it to decide who is going to get the treatment or what roles you are you going to play?** (Please select **ONE** correct answer from “1” to “5”)

1. □ Very hard
2. □ Hard
3. □ Neither hard, nor easy
4. □ Easy
5. □ Very easy

**3D - What were the criteria to define who gets the treatment (or roles, in case of ROPA - decide who is the donor and who is the recipient)?** (select **ALL** the correct answers))

a. □ random
b. □ age
c. □ one of you has a contraindication for pregnancy
d. □ one of you has a condition that affects fertility
e. □ one of you has a stronger desire to get pregnant
f. □ preference for transmitting the genetics of one of you.
g. □ one of you has a genetic alteration and you wanted to avoid its transmission
h. □ same as previous treatment, so your children can share genetics.
i. □ opposite compared to previous treatment, so that both can play an active role / both roles.
j. □ treatment at the same time, so that you can both actively participate simultaneously.
k. □ other:_________________________________________

### 4 - IMPORTANCE OF PREGNANCY AND GENETICS

(Please select **ONE** correct answer for each question)

**4A - How would you rate your WISH to be a mother?**

1. □ Very low
2. □ Low
3. □ Moderate
4. □ High
5. □ Very high

**4B - How important is it to you to be PREGNANT of your children?**

1. □ Not at all important
2. □ Slightly important
3. □ Moderately important
4. □ Very important
5. □ Extremely important

**4C - How important is it to you that your children have your GENES?**

1. □ Not at all important
2. □ Slightly important
3. □ Moderately important
4. □ Very important
5. □ Extremely important

**4D - How ATTACHED do you think your CHILD will likely be to you and your partner?**

□ Probably more attached to me

□ Equally attached to both

□ Probably more attached to your partner

### 5 - FUTURE

**5A - How would you rate your wish to have MORE CHILDREN after this treatment?** (Please select **ONE** correct answer)

1. □ Very low
2. □ Low
3. □ Moderate
4. □ High
5. □ Very high

**5B - Imagine that you are going to have a NEW CHILD after this treatment, which of the following would be your first option?**

(Please select **ONE** correct answer from “a” to “f”) Artificial insemination (intrauterine) / *in vitro* fertilization (IVF)

a. □ You would get the treatment
b. □ Your partner would get the treatment
c. □ Both would have the treatment ROPA (shared IVF)
d. □ Genetic mother (you would donate the eggs; your partner would get pregnant with your eggs)
e. □ Gestational mother (you would get pregnant with your partner’s eggs)
f. □ Reciprocal ROPA: exchange of eggs and both would get pregnant with the same treatment
